# Minimal invasive single-site surgery in colorectal procedures: Current state of the art

**DOI:** 10.4103/0972-9941.72382

**Published:** 2011

**Authors:** Michele Diana, Parag Dhumane, R A Cahill, N Mortensen, Joel Leroy, Jacques Marescaux

**Affiliations:** Department of Surgery, IRCAD/EITS, Hôpitaux Universitaires, 1 Place de l’Hôpital, 67091, Strasbourg Cedex, France; 1 Department of Surgery, Radcliffe Hospitals, Oxford, United Kingdom

**Keywords:** Colorectal surgery, laparoendoscopic single-site surgery, minimal invasive single-site surgery, single-incision laparoscopic surgery, single port access

## Abstract

**BACKGROUND:**

Minimally invasive single-site (MISS) surgery has recently been applied to colorectal surgery. We aimed to assess the current state of the art and the adequacy of preliminary oncological results.

**METHODS:**

We performed a systematic review of the literature using Pubmed, Medline, SCOPUS and Web of Science databases. Keywords used were “Single Port” or “Single-Incision” or “LaparoEndoscopic Single Site” or “SILS™” and “Colon” or “Colorectal” and “Surgery”.

**RESULTS:**

Twenty-nine articles on colorectal MISS surgery have been published from July 2008 to July 2010, presenting data on 149 patients. One study reported analgesic requirement. The final incision length ranged from 2.5 to 8 cm. Only two studies reported fascial incision length. There were two port site hernias in a series of 13 patients (15.38%). Two “fully laparoscopic” MISS procedures with preparation and achievement of the anastomosis completely intracorporeally are reported. Future site of ileostomy was used as the sole access for the procedures in three studies. Lymph node harvesting, resection margins and length of specimen were sufficient in oncological cases.

**CONCLUSIONS:**

MISS colorectal surgery is a challenging procedure that seems to be safe and feasible, but the existing clinical evidence is limited. In selected cases, and especially when an ileostomy is planned, colorectal surgery may be an ideal indication for MISS surgery leading to a no-scar surgery. Despite preliminary oncological results showing the feasibility of MISS surgery, we want to stress the need to standardize the technique and carefully evaluate its application in oncosurgery under ethical committee control.

## INTRODUCTION

Minimizing the insults to the abdominal wall is intuitively accompanied by improved outcomes and, indeed, the laparoscopic approach in colorectal surgery has proven its benefits over open surgery in terms of lower postoperative pain, faster recovery and better cosmetic results.[1–3] The rationale for further scar-less surgery is that countdown in number and size of port access to the abdominal cavity may be advantageous not only from the cosmetic aspect but also in order to minimize the risk of complications such as wound pain and infections as well as incision hernia and internal adhesion formation.[4]

The ultimate form of low-impact surgery is likely to be the use of natural orifices to reach visceral organs and thereby completely avoiding abdominal wall incisions. Challenges encountered during technological development of Natural Orifice Transluminal Endoscopic Surgery (NOTES™) however have given an important impulse to the recent introduction and impetus of the single-access laparoscopic surgery. Passing all necessary laparoscopic instruments through a single small incision, especially when this is sited at the natural umbilical scar, allows the execution of a virtual NOTES™ procedure with minimal parietal trauma. Although first practiced in the early 1990s (Begin reported the first single-incision laparoscopic appendectomy in 1993[5] while Navarra performed the first single-incision laparoscopic cholecystectomy in 1995),[6] technical difficulties have hampered its widespread implementation until recently. In the past 3 years, compelling research in minimal invasive surgery and important technological improvements have marked the “rebirth” of the single-wound approach, which is now rapidly gaining the favour (and curiosity) of the surgical community. The feasibility of this minimal invasive technique has now been assessed in several digestive procedures, with large published series of cholecystectomies,[7–10] appendectomies[11] and bariatric procedures.[12]

There is of course a plethora of acronyms in the literature referring to single-site surgery such as SILS™ (single-incision laparoscopic surgery), SPA™ (single-port access), SPS (single-port surgery), NOTUS (natural orifices transumbilical surgery), eNOTES (embryonic natural orifices transluminal endoscopic surgery) and SAES (single-access endoscopic surgery) as well as others that are at least as confusing.[13] Recently, a consensus statement of the consortium for LaparoEndoscopic Single-Site Surgery proposed the acronym of LESS as the official denomination, although we have the feeling that using “LESS” might have commercial concerns.[14] Furthermore, because this surgical concept could be extended even to nonabdominal minimal invasive procedures, e.g. video-assisted thoracoscopies or thyroidectomies, we propose the use of minimal invasive single-site (MISS) surgery to include a broader spectrum of surgeries and we will use it through this article.

The main difference to retain is between “single-incision surgery,” in which a single skin incision is made and ports pass individually through multiple fascial incisions and “single port surgery,” in which instruments are passed into a unique port into a single fascia wound. In MISS, therefore, all instruments come from a unique entry point with almost parallel orientation. This raises a number of specific new challenges compared with the laparoscopic conventional approach. A reduced capacity for triangulation is the most claimed issue, with its imposed need to operate sometimes with crossed hands. Furthermore, the repeated conflicts between the shafts of the instruments, the gastroenterologist-like in-axis view offered by the telescope running parallel to operating instruments and the difficulties to achieve a correct exposure and to apply the necessary traction to tissues without a supplementary instrument all complete the list of the major problems. The use therefore of this new approach for complex colorectal procedures implying multiple-field dissections, resection and retrieval of huge specimens as well as for the construction of anastomosis could therefore understandingly be viewed as difficult to implement, especially for oncological cases.

MISS has however been applied to colorectal surgery in the 2 years since Professor Leroy described and standardized the first single-port sigmoidectomy[15] and performed the first totally laparoscopic MISS sigmoidectomy with endoluminal placement of the anvil of the circular stapler to allow intracorporeal anastomosis.[16] In a review published this year, Leblanc *et al*.[17] have reported data on 17 patients from nine available studies and even since then, due to the high level of interest in this field, more and more reports and small series have been published. Most recently, the Oxford group have published the first series of MISS total colectomy for patients with medically uncontrolled colitis, which involves a standardized operative approach specifically formatted to supervene the limitations of the confined operative approach.[18]

Our aim here is to benchmark the current state of the art of MISS on colorectal surgery, analysing clinical reports and case series, paying attention to initial operative outcomes and oncological preliminary results.

## MATERIALS AND METHODS

We performed a systematic review of the English literature using the databases Pubmed, SCOPUS, MEDLINE and WEB of SCIENCE since July 2008. Keywords used were “Single Port” OR “Single-Incision” OR “Laparoendoscopic Single-Site” OR “SILS™” AND “colon” OR “Colorectal” AND “Surgery” in various combinations. Single-access or single-port appendectomies were not included in our review. All pertinent articles were carefully analyzed by MD, PD and JL.

## RESULTS

From July 2008 to July 2010, a total of 29 articles and one systematic review have been published in the English literature on single-access laparoscopic colorectal surgery. Fourteen technical notes with one patient and 14 case series (one series[19] being the extension of a previous published one[20]) were analysed. One series in robotic single-incision colorectal surgery has been published.[21] One ongoing randomized clinical trial has been identified. (Trial for single port versus conventional laparoscopic colectomy. “ClinicalTrials.gov” Identifier NCT01101672.) Therefore, a total of 149 patients (121 in MISS with single-port device and 28 in MISS without single-port device) have been reported by the time of the present review. The diagnostic and surgical procedure lists are shown in Tables 1 and 2. In contrast, the last published systematic review on colorectal single-access surgery displayed data concerning 17 patients.[17] Outcomes in single-incision cases are displayed in Table 3. Tables 4 and 5 summarize the technical aspects and operative outcomes of MISS with single-port procedures, respectively. Results concerning the number of harvested nodes, specimen length and resection margins in the case performed for malignancy are displayed in Table 6.

**Table 1:** Diagnostics

	n°
Colorectal Cancer	91
Diverticular disease	28
Polyps	13
Inflammatory bowel disease	11
Slow transit constipation	2
Endometriosis	2
Familiar Adenomatosis Polyposis	1
Lymphoma	1
	149

**Table 2:** Therapeutic Operations

	n°
Right Colectomy	91
Sigmoidectomy	30
Total Proctocolectomy	6
Left Colectomy	6
Total Colectomy	10
Anterior Resection	3
Low Anterior Resection + TME	3
Ileo-colic Resection	2
Abdominoperineal Resection	1
	149

**Table 3:** MISS without single port devices procedures

	Operation N	Trocars (mm)	IL (cm initial/final)	Op time (min)	Complications	LOS days (range)
Brunner[45]	Sigm = 2	5-5-5	2/2	110 and 180	none	7 and 6
Rieger[46]	Rt Col = 6, Left Col = 1	12-5-5	3,1 (2,5-4,5)**/ ns	89 (75-115)**	1 bacteremia	5,4 (4-11)
Podolsky[25]	Sigm = 8	5-5-5	1,8/ns	169§	2 incision hernia	5-8
	Rt Col = 3	5-5-5	1,8/ns	155§	1 wound infection	6-7
	Left Col =1	5-5-5	1,8/ns	179		5
	Tot proct =1	5-5-5	1,8/ns	300		5
Takemasa[47]	Sigm = 1	5-5-5	2/2	192	none	7
Bucher[48]	Sigm = 1	12-5	2/2	125	none	2
Bucher[49]	Left Col = 1	12-5	2/2	213	none	n.s.
Bucher[50]	Rt Col = 1	12-5	3/3	158	none	n.s.
Osterwitz[21]	Rt Col = 2	12-8-8	4/4	132 and 158	none	4 and 3
	Tot = 28					

** = median;

Ns = not specified; IL = incision length; LOS = length of hospital stay; Sigm = sigmoidectomy; Rt Col= Right Colectomy; Tot proct = Total proctocolectomy

**Table 4:** Technical aspects of MISS with single port devices procedures

Procedure	PORT TYPE n patients (105)		SCOPE	Instruments	Exposure	Vessel Ligation	Anastomosis	Fully Laparoscopic
Right Colectomy (n=75)	SILS™ (n total = 36)	21[22]	n.s	Roticulating	n.s.	L, U. Bipolar, clips	EC; Hs; n.s.	NO
		13[24]	n.s	n.s.	n.s.	n.s.	EC; S; S-S	NO
		1[21]	n.s.	n.s.	n.s.	L	EC; S; E-S	NO
		1[32]	30° Standard	Roticulating	Transparietal suture	L	IC, S; S-S	YES
		10[30]	30° Standard	Roticulating	Grasper		EC, S, E-E	NO
	Triport™	6[26]	5 mm FT §	n.s.	n.s.	L, S, H	EC; S; n.s.	NO
	Endocone™	15[22]	n.s.	Curved	n.s.	L, U. Bipolar, clips	EC; Hs; n.s.	NO
	Uni-X™	1[23]	5 mm FT	Curved	n.s	L	EC; S; E-S	NO
	Gelport™	11[51]	30° Standard	Standard	n.s.	Stapling device	EC; n.s.	NO
	Revolving multiport device	5[29]	30° Standard	n.s	EC magnetic retraction	n.s.	EC; n.s.	NO
	One-glove technique	1[52]	0° Standard	Standard	n.s	L	n.s	NO
Sigmoidectomy (n=18)	SILS™	10[28]	30° Standard	Roticulating	n.s	L, S, H	IC; S; ns	NO
	Revolving multiport device	5[27]	30° Standard	n.s.	Rolled Gauze	n.s.	IC; S; n.s.	NO
	Triport™ (n total = 2)	1[16]	0° Standard	Roticulating	Magnetic Anvil	L	IC; S; E-E	YES
		1[53]	n.s.	n.s.	n.s	L	IC; S; n.s.	NO
	One-glove technique	1[34]	0° Standard	n.s.	n.s.	n.s.	IC, S; S-E	NO
Left Colectomy (n=3)	Revolving multiport device	2[29]	30° Standard	n.s.	EC magnetic retraction	n.s.	EC; ns	NO
	Triport™	1[54]	5 mm FT	n.s.	Transabdominal suture	S;H	EC; HS; E-E	NO
Proctocolectomy (n=4)	SILS™	1[35])	5 mm FT	n.s.	n.s.	L	IC, S, E-E	NO
Total Colectomy (n=4)	Gelpoint™	1[55]	5 mm FT	Flexible grasper	n.s.	L	IC, S, E-E	NO
	SILS™	3[18]	30° Standard	Standard	Standard instruments	L	None***	NO
Anterior Resection (n=1)	Triport™	1[26]	5 mm FT	n.s.	n.s.	L, S, H	IC, S, E-E	NO

Scope: FT Flexible Tip; Approach: -MtL: medial to lateral. LtM: Lateral to medial. Vessel ligation: -Ligasure™ (L); -Ultracision™ (U);-Sonosurg™ (S);-Hemolock™ (H); Anastomosis: -Intra-extracorporeal (IC, EC); -Stapled-Hand sewn (S, Hs);-Orientation E-E: end-to-end, S-S: side-to-side; S-E: side-to-end.

*** (first of three stage proctocolectomy with ileoanal pouch); SILS™ Covidien Mansfield MA USA; Triport™ Advanced Surgical Concepts, Wicklow Ireland; Gelpoint™ and Gelport™ Applied Medical, Rancho Santa Margarita CA; Endocone™ Karl Storz GmbH Tuttlingen Germany; Uni-X™ Pnavel Systems Morganville NJ USA; LigaSure™ Covidien Valley Lab Norwalk Connecticut; Ultracision™ Ethicon Endosurgery Cincinnati OH USA; SonoSurg™ Olympus America Inc; Hemolock™ (Teleflex Inc)

**Table 5:** Operative outcomes in MISS with single port devices

Procedure (tot) -Ref	Op. time (min) (range) # = median		Incision lenght (cm) Initial/Final/Range			Conv	Complications Number Dindo Score[31]	LOS Days (range)	
Right Colectomy N = 79									
Boni[22] n=36	145 ± 21	-	3,5	3,5	n.s.	0	2/36 Dindo II	5 ± 1,2	4-14
Ramos-V[24] n=13	131,5 ± 36,2	79-180	2,5	n.s.	2,5-6	2	1/13 Dindo II	2,5 ± 0,7	2-4
Law[26] n=6	175#	103-260	3,4	n.s.	3-5	1	1/6 Dindo II	3,5	3-6
Uematsu[29] n=5	255# §	220-315	3-4	n.s.	n.s.	0	0	7	-
Gash[19] n=4	66,5#	45-105	2,5	n.s.	n.s.	0	n.s.	1	0,5-1,91
Ostrowitz[21] n=1	166		2-3	4		1	0	4	
Wong[30] n= 10	83#	60-125	2,5	5#	4-8	0	0	6	5-11
Remzi[23] n=1	115		3,5	n.s.		0	0	4	
Keshava[51] n=1	105		3	n.s.		0	0	3	
Choi[52] n=1	180		3	n.s.		0	0	4	
Morales-Conde n=1	140		2,5	2,5		0	0	4	
Sigmoidectomy N = 18									
Vestweber[28] n=10	120#	85-156	2,5	n.s.	n.s.	1	1/10 Dindo I	7#	6-15
Uematsu[27] n=5	185#	176-210	3-4	4-5	n.s.	1	0	7	
Leroy[16,34] n=2	90		2,5	2,5		0	0	4	
Remzi[53] n=1	198		3	n.s.		0	0	3	
Left Colectomy N = 3									
Uematsu[29] n=2	255#	220-315	3-4	n.s.	n.s.	0	0	7	
Law[54] n=1	180		3	n.s.		0	0	3	
Proctocolectomy N = 5									
Geisler[35] n=1	172		2	2		0	0	4	
Gash[19] n=4	177,5#	130-195	2,5	n.s.	n.s.	1	n.s.	3,2#	2,3-9
Total Colectomy N = 7									
Bardakcioglu[55] n=1	n.s.		3	n.s.		0	0	4	
Gash[19] n=3	110#	58-130	2,5	n.s.	n.s.	0	n.s.	2,5	1,125-2,5
Cahill[18] n=3	206	177-235	2,5	2,5		0	1/3 Dindo IIIb	5,1	3-8
Anterior Resection N = 6									
Law[26] n=1	175#	103-260	3	n.s.		0	0	3	
Gash[19] n=5	110#	75-210	2,5	n.s.	n.s.	0	n.s.	0,8	0,66-2,66
Ileocolic Resection N= 2									
Gash[19] n=2	45, 240	45-240	2,5	n.s.	n.s.	0	n.s.	7,16	7-16
Abdominoperineal excision N=1									
sh[19] n=1	135		2,5	n.s.	n.s.	0	n.s.	6	

**Table 6:** Miss for colorectal malignancy

Author	N Tot = 91	Location of tumor	Harvested LFN	Specimen lenght cm	Resection margins cm (prox/dist)
Rieger[46]	7	6 Right Colon 1 Splenic Flexure	15 (7-26)	n.s.	Adequate n.s.
Ramos-V[24]	13	13 Right Colon	26,7 ± 14,5 (14-47)	22,1 ± 7 (11-33,7)	Negative n.s.
Remzi[53]	1	1 Sigmoid	14	16	10/5,5
Podolsky[25]	8	3 Right Colon 1 Left Colon 4 Sigmoid	12-18 14 13-16	n.s.	Negative n.s.
Bardakcioglu[55]	1	Synchronous caecum and sigmoid	17	n.s.	n.s
Takemasa[47]	1	1 Sigmoid	14	20	Negative n.s
Law[26,54]	5	3 Right Colon 1 Transverse Colon 1 Upper rectum	13,5** (9-36)	27,5** (11,5-43)	Negative n.s
Uematsu[27,29]	12	5 Sigmoid	17**	n.s.	n.s.
		2 Descending Colon 5 Ascending Colon	32** (15-38)		
Boni[22]	32	32 Right Colon	24 ± 7 (29-15)	n.s.	8 ± 3 (6-12)
Choi[52]	1	1 Right Colon	69	27,5	10
Ostrowitz[21]	1	1 Right Colon	22	n.s.	7
Bucher[49]	1	1 Left Colon	34	39	10
Morales-Conde[32]	1	1 Right Colon	15	Ns	Negative
Wong[30]	8	4 Ascending Colon	16 (10-19)	Ns	Negative
		2 Caecum 1 Mid transverse			
		1 terminal ileum (Lymphoma)			

** = median;

Ns = not specified

The main points of interest are listed here. No studies reported use of the postoperative Visual Analogic Scale of pain. Analgesic requirement was however reported in the largest series of right hemicolectomies.[22] The maximum span of follow-up in colorectal procedure was 1 year.[23] Body mass index higher than 25 kg/m^2^ was identified as a factor of operative laboriousness, significantly affecting operating time.[24] Podolsky *et al*.[25] reported two cases of incisional hernia in their series of 13 patients (15.38%). In the subgroup of MISS procedures with single-port devices, 5/121 conversions are reported: three to hand assisted,[24,26] one to multiple ports,[27] one to open procedure.[28] There were two conversions to single port in the robotic-assisted MISS, right hemicolectomies due to lack of tight air-seal with consequent loss of pneumoperitoneum.[21] Initial skin incision length was reported in all studies, which ranged from 1.8[25] to 3–4 cm[22,27,29] till 6 cm,[24] but the final skin incision length was clearly expressed in 12/29 studies as going up to 8 cm.[30] In few studies, fascia incision length was reported,[27,29] which ranged from 4 to 5 cm. Beside the cases in which conversions occurred.[24,26] only one study[18] reported one major complication intended as Dindo Score ≥3.[31] Only three MISS “fully laparoscopic” procedures are published:[16,32,33] we reported the first fully laparoscopic sigmoidectomy[16] and, recently, Morales-Conde *et al*.[32] performed the first fully laparoscopic MISS right hemicolectomy with stapled intracorporeal anastomosis using the Endostitch suture system (Covidien, Norwalk, CT) to close the intestinal breach.

## DISCUSSION

In 2008, we demonstrated the feasibility and reproducibility of single-port sigmoidectomy in a porcine survival model involving six pigs.[15] The passage from bench to bed has been very rapid, with an increasing number of clinical reports of MISS in colorectal surgery. The complexity of colorectal surgery however makes more apparent the already recognized technical difficulties of single-access surgery. To summarize these aspects, the lack of triangulation and exposure, the in-axis view and the repeated conflicts between instruments are the most important challenges. A large variety of tricky and inventive solutions to overcome these difficulties has already been displayed in these preliminary studies, like the use of curved or roticulating instruments, sleeve ports, flexible tip scopes, magnetic retraction, intracorporeal fixing devices and/or the use of a robotic interface. An extensive analysis of technological unresolved issues is however beyond the aim of this review. Furthermore, these studies are highly inhomogeneous where technical aspects are concerned, showing that the technique is still in its infancy, and most of them present a lack of certain outcome measures that deserve to be discussed.

If one of the expected advantage of MISS surgery is the reduction of postoperative pain, one may be surprised that none of these reports assessed the postoperative pain using a validated tool such as the Visual Analogue Scale, and only in one did we find specifications concerning the analgesic requirement.[22] Concerning the incision length, it would be of interest to specify systematically the length of the fascia incision that is often enlarged to permit the extraction of the specimen. It has recently been reported that a mid-line extraction site of the specimen in laparoscopic colorectal surgery greatly increases the risk of incision hernia compared with off-midline sites (midline 17.6% vs. off-midline 7.8%; *P* < 0.0002).[34] Podolsky *et al*., using the technique named SPA surgery reported two access site hernias in their series of 13 colorectal procedures (15.38%).[25] SPA implies the realization of three to four distant fascia incisions along with the mobilization of soft tissue flaps off the underlying fascia to create a quite large working space in which separate trocars are placed, offering a good triangulation. At the time of extraction of the specimen to place the anvil and/or perform an extracorporeal anastomosis, fascia incisions are connected, with the consequence of the creation of a point of potential weakness. Performing an extracorporeal anastomosis within a very small incision may be not only cumbersome but also traumatic for the colon and mesocolon; however, enlarging it too much may lead to a loss of the intended benefits. On the other hand, the conversion to hand-assisted laparoscopy with a 6-cm incision is probably to be considered out of the setting of minimally invasive surgery and, more realistically, be seen as a “video assisted minilaparotomy.” It should of course be stated that the publications to date are initial experiences and therefore perhaps best viewed as proof of feasibility rather than proof of benefit.

The publication base to date does however point to the technical difficulties and raises issues that need consideration and address for this operative approach to progress fully into mainstay clinical practice. To solve this issue of extracorporeal extraction for the purposes of left-sided resections, our preference is to perform a “fully laparoscopic” MISS sigmoidectomy with endoluminal placement of the anvil of the circular stapler to prepare and perform the anastomosis completely intracorporeally without exteriorization of the bowel, with the retrieval of the specimen either transanally or transumbilically performed at the very end of the procedure.[16] More recently, we have also presented an original technique of percutaneous anvil positioning that may facilitate colorectal full-laparoscopic MISS surgery in the same philosophy of optimizing the technique.[33] This technique prevents from loss of working space and shortens the operating time, and is a notable step toward hybrid NOTES™, procedures as outlined also by Morales.[32] To completely avoid enlargement of the fascia incision, the transanal specimen extraction is a feasible, safe and effective solution to reduce the risk of access site hernia, especially in nononcological cases, provided it does not increase infectious complication risks.[33] Another elegant solution that makes application of MISS to colorectal surgery very appealing is the possibility of using the future site of a planned ileostomy as the “entry” and “exit” point with a minimal parietal trauma and a virtual no-scar procedure.[18,19,35] This is particularly facilitated if specimen bulk is minimized by performing the dissection in a close pericolic plane, as is possible in benign cases.[18] In addition, such a plane avoids major vessels and allows the energy device used for dissection to fully seal the small segmental, branching vessels that feed directly into the colon.[18] In cases in which no ileostomy is planned and a transanal extraction is not feasible, transumbilical trauma can be minimized if the surgeon renounces a strict MISS procedure and uses a less-traumatic incision, such as the Pfannenstiel incision, to prepare the anastomosis and extract the specimen along with the use of very low-profile 5 mm trocars. Because cosmesis is one of the most important arguments in favour of MISS surgery, one may speculate about a possible threshold of skin incision length to remain in this setting and also about the most suitable shape and orientation of the incision to offer better results and lower parietal trauma. We actually lack objective tools to assess cosmetic results or body image disturbance because of the scar in this specific surgery, and the authors rely on expressed satisfaction of patients. Incision must be adapted to the anatomy of the patient and to the type of operation, and cannot be standardized. The shape may be longitudinal or vertical, or even in “reverse smile,” as proposed by Boni, and may pass either directly through the umbilical scar or just laterally and oriented on the opposite of the surgical field. Comparisons concerning the shape of the skin incision may be the focus of further investigations.

The robotic interface may in future also overcome several limits of the single-port setting, as displayed in experimental models.[36]

Furthermore, especially when a transanal extraction of the specimen is planned, a combined transanal access using as platform either the Transanal Endoscopic Operation system (TEO™, Karl Storz^®^ Germany) or the new-generation operating scopes (ISISSCOPE Karl Storz^®^ Germany) may fulfil the lacunae we currently face with pure transumbilical MISS in terms of limited traction or restricted instrumentation. In the same spirit, very low-profile MISS instruments may provide assistance to perform NOTES™ transanal colorectal procedures during this transitional phase. Since its introduction, MISS surgery has been regarded as an alternative to NOTES™ in the panorama of minimal invasive surgery. We believe more in the concept of “integration” between these two technologies and philosophies. Probably, the best instrument set is the combination of rigid and flexible technology with straight and curved instruments with different sizes that permit to achieve a better triangulation, and a sensible next step would be the introduction of a flexible or articulated clip applier and/or an articulated thermal sealing device for vascular control and dissection.

Some considerations are also necessary concerning the use of an MISS approach and oncological colorectal surgical pathology. As we all know, the factor with the highest impact on the natural history is the early diagnostic policy with the possibility to deal with early-stage cancers. There is a broad spectrum of therapeutic possibilities for colorectal cancer, ranging from endoscopic procedures like the endoluminal piecemeal mucosal resection (EPMR) or, more recently, endoscopic submucosal dissection (ESD), passing through full-thickness resection in early-stage rectal cancers using the Transanal Endoscopic Microsurgery (TEM) platform,[37–39] to conventional laparoscopic surgery and ultimately to laparotomy. A recent metaanalysis has demonstrated adequacy of oncological results of laparoscopy compared with the open approach in colorectal surgery with similar disease-free and overall long-term survival rates.[2] The adequacy of lymph node retrieval plays an important role in tumour staging and prognosis,[40] and factors influencing the number of resected nodes are many. Laparoscopy is as effective as open surgery in accomplishing this task.[41] Node retrieval and specimen length in MISS surgery are comparable to multiport laparoscopic results in these preliminary data, but given the limited number of patients and the shortness of follow-up, no conclusions might be drawn. Furthermore, concerning the risk of port site metastasis, if intuitively the reduction of number of ports reduces the number of risky sites, the higher difficulties to respect a “no touch” technique with rigorous oncosurgical principles must be considered when making this kind of a reflection. We feel that the retrieval of a colon tumour could not be performed through a small incision of 1.8 cm because the surgical manipulation and the strangulation of the feeding mesentery may damage the splancnic endothelium and lead to local or even portomesenteric venous thrombosis formation.[42] Concerning selection criteria for oncological patients to undergo MISS, these are far to be determined but probably large tumours might be excluded. Regarding resection margin adequacy, a more systematic pathological report including proximal and distal resection margin would be important to make long-term comparisons. Despite this very preliminary data, we believe that MISS evolution could be of unexpected value in the future of colorectal surgery, with standard laparoscopy being a backup procedure. Indeed, MISS may represent the nodal link between robotic, NOTES™ and laparoscopy.

Development and standardization of surgical platforms and techniques offering less-invasive and equally effective procedures is the common aim of physicians and engineers of surgical companies. Immediate reaction to novelties is normally a mix between enthusiasm and scepticism. Both reflect a merely intuitive appreciation without the whole vision of the possible implications. Especially in the surgical field, given the complex interactions between different actors (the patient, the surgeon, the companies, the health care system), the process of acceptance or rejection of a new technique is extremely delicate. Examination of this issue from different points of view may better explicate the concerns and challenges posed by this technique.

Patient’s fears are mainly concentrated on postoperative pain and possible complications, and also on body image change and quality of life. Having the choice of course patients prefer minimal invasive techniques and, among the possible options, including conventional multiport laparoscopy, single-access surgery and NOTES™, the most appealing in a well-conducted survey was found to be MISS surgery.[43] Particularly interesting is the fact that one-third of the 750 participants would accept single-port surgery or NOTES™ even without a proven safety profile and that 80.6% preferred the single-port approach to NOTES™.

This wide acceptance of MISS converts into a high potential for developing the technique, driven by patient demand, and surgeons must be prepared and feel the urge to improve feasibility and safety of this approach. On the other hand, surgical instrument and technology companies are looking for establishing a convenient and competitive market. This is absolutely normal and positive results and improvements are possible only with tight and ethical cooperation with companies. Finally, health care systems have the duty to offer to the citizen the best of medical care, taking into account the economic aspect. This novel approach therefore has to prove a favourable cost/benefits ratio to gain widespread acceptance.

Surgeons are in the midst of this storm of competing interests. Between the natural tendency to explore new frontiers and the need to offer evidence-based results, surgeons will be the main decision makers at the crossroads of the interests of patients, companies and health care systems. Patients’ desire may therefore not always be accomplished and sometimes should even be discouraged when appropriate selection criteria are not fulfilled. Pressure of companies must be weighted with the need of a scientific approach. Considering the need of important fund allocations to ameliorate current instrumentation, a comprehensive cost/benefits analysis is mandatory. If we may tolerate that the introduction of laparoscopic cholecystectomy (which has doubled the risk of biliary duct injuries compared with open surgery[44]) is because of the overwhelming global benefits of laparoscopy, could we stand a further increase of complications without the proof of a similar outstanding advantage? Actually, the available hard facts concerning patient’s outcomes do not as yet permit any conclusions.

## CONCLUSIONS

MISS colorectal surgery is a challenging procedure that seems to be safe and feasible, but currently actual experience in significant numbers of patients is very limited. Furthermore, the existing clinical evidence base is highly inhomogeneous and follow-up data are limited. Nonetheless, it is becoming clear that in selected cases and especially when an ileostomy is planned, colorectal surgery may be an ideal indication for MISS surgery leading to a no-scar surgery. However, even if these very preliminary results in the colorectal oncological pathology seem to show the feasibility of MISS surgery, we want to stress the need to standardize the technique and carefully evaluate this application under the control of dedicated ethical committees. MISS should therefore be included in the armamentarium of the modern colorectal surgeon because it could represent the link between NOTES™, robotic and laparoscopic surgery. Improvement of instrumentation will probably enhance its widespread diffusion but cost analysis and randomized clinical trials are clearly needed.
